# Novel Factors of Viral Origin Inhibit TOR Pathway Gene Expression

**DOI:** 10.3389/fphys.2018.01678

**Published:** 2018-11-26

**Authors:** Rosanna Salvia, Marisa Nardiello, Carmen Scieuzo, Andrea Scala, Sabino A. Bufo, Asha Rao, Heiko Vogel, Patrizia Falabella

**Affiliations:** ^1^Department of Sciences, University of Basilicata, Potenza, Italy; ^2^Department of Biology, Texas A&M University, College Station, TX, United States; ^3^Department of Entomology, Max Planck Institute for Chemical Ecology, Jena, Germany

**Keywords:** Polydnavirus, *Tn*BV, PI3K/Akt/TOR, ecdysteroidogenesis, prothoracic glands

## Abstract

Polydnaviruses (PDVs) are obligate symbionts of endoparasitoid wasps, which exclusively attack the larval stages of their lepidopteran hosts. The Polydnavirus is injected by the parasitoid female during oviposition to selectively infect host tissues by the expression of viral genes without undergoing replication. *Toxoneuron nigriceps* bracovirus (*Tn*BV) is associated with *Toxoneuron nigriceps* (Hymenoptera: Braconidae) wasp, an endoparasitoid of the tobacco budworm larval stages, *Heliothis virescens* (Lepidoptera: Noctuidae). Previous studies showed that *Tn*BV is responsible for alterations in host physiology. The arrest of ecdysteroidogenesis is the main alteration which occurs in last (fifth) instar larvae and, as a consequence, prevents pupation. *Tn*BV induces the functional inactivation of *H. virescens* prothoracic glands (PGs), resulting in decreased protein synthesis and phosphorylation. Previous work showed the involvement of the PI3K/Akt/TOR pathway in *H. virescens* PG ecdysteroidogenesis. Here, we demonstrate that this cellular signaling is one of the targets of *Tn*BV infection. Western blot analysis and enzyme immunoassay (EIA) showed that parasitism inhibits ecdysteroidogenesis and the phosphorylation of the two targets of TOR (4E-BP and S6K), despite the stimulation of PTTH contained in the brain extract. Using a transcriptomic approach, we identified viral genes selectively expressed in last instar *H. virescens* PGs, 48 h after parasitization, and evaluated expression levels of PI3K/Akt/TOR pathway genes in these tissues. The relative expression of selected genes belonging to the TOR pathway (*tor*, *4e-bp*, and *s6k*) in PGs of parasitized larvae was further confirmed by qRT-PCR. The down-regulation of these genes in PGs of parasitized larvae supports the hypothesis of *Tn*BV involvement in blocking ecdysteroidogenesis, through alterations of the PI3K/Akt/TOR pathway at the transcriptional level.

## Introduction

*Toxoneuron nigriceps* (Viereck) (Hymenoptera: Braconidae) is a solitary braconid endoparasitoid wasp of the tobacco budworm *Heliothis virescens* (Fabricius) (Lepidoptera: Noctuidae) larval stages. During oviposition, *T. nigriceps* female injects several maternal factors into the host (Lewis and Vinson, 1968; Pennacchio et al., 1993), which are able to modulate the host immune system and endocrine balance, in order to create a suitable environment for parasitoid progeny development (Beckage and Gelman, 2004; Pennacchio and Strand, 2006; Falabella et al., 2007; Moreau and Asgari, 2015). Maternal factors include protein rich venom (Laurino et al., 2016) and the ovarian calyx fluid, composed of ovarian proteins and an obligate symbiotic virus belonging to the Polydnaviridae family, the *Toxoneuron nigriceps* bracovirus (*Tn*BV) (Lewis and Vinson, 1968). The genome of Polydnaviruses (PDVs) consists of multiple double-stranded segmented circular DNA, integrated as provirus into the wasp genome, specifically associated in a mutualistic symbiosis (Webb et al., 2000; Webb and Strand, 2005). PDVs integrated into the parasitoid genome replicate exclusively in wasp ovaries, where their circular genome is generated from linear DNA copies of insect chromosomes (Varricchio et al., 1999; Volkoff et al., 2010; Dupuy et al., 2011). PDVs express their genes, without any replication event, in several host tissues, causing alterations in their physiology and enabling parasitoid larvae to survive, grow and finally pupate in silken cocoons (Strand and Burke, 2014). Thus, successful development of parasitoid wasp ensures vertical transmission of the integrated viral genome to the next generation (Strand, 2010).

In *H. virescens* larvae parasitized by *T. nigriceps*, the main infection target tissues are haemocytes and prothoracic glands (PGs) (Stoltz and Vinson, 1979; Wyder et al., 2003), where *Tn*BV expresses several genes belonging to different families encoding protein tyrosine phosphatases (PTPs), the largest gene family in bracovirus (Provost et al., 2004; Falabella et al., 2006, 2007; Webb et al., 2006), ankyrin motif protein (ANK) (Falabella et al., 2007; Salvia et al., 2017), UDP dehydrogenase, proteins containing Ben domains (Park and Kim, 2010; Falabella et al., unpublished data). Other viral genes expressed in the host are *Tn*BV1, implicated in immune suppression (Varricchio et al., 1999; Malva et al., 2004) and activating caspase proteins (Lapointe et al., 2005) and *Tn*BV2 encoding a retroviral aspartyl protease (Falabella et al., 2003). Previous studies reported that *H. virescens* PGs explanted from parasitized last (fifth) instar larvae do not respond to the stimulus of the prothoracicotropic hormone (PTTH), the neuropeptide that triggers ecdysone production (Pennacchio et al., 1997, 1998a). Besides the inhibition of ecdysteroidogenesis, a decrease of protein synthesis and a complete inhibition of PTTH-induced phosphorylation of some unidentified proteins in PGs of parasitized larvae were observed (Pennacchio et al., 1997, 1998a*)*. Moreover, it was demonstrated that *Tn*BV is responsible for the functional inactivation of PGs and thus for blocking host pupation (Pennacchio et al., 1998a) due to the reduction of ecdysone biosynthesis in parasitized last instar larvae. Recent work reported that the TOR pathway is involved in ecdysteroidogenesis in some lepidopteran species (Gu et al., 2012; Kemirembe et al., 2012), including *H. virescens* (Scieuzo et al., 2018).

In this work, we show that specific *Tn*BV genes are expressed in PGs of *H. virescens* last instar larvae and we argue that they could be involved in blocking ecdysteroidogenesis, at least in part, through negative regulation of the PI3K/Akt/TOR pathway at the transcriptional level.

## Materials and Methods

### Insect Rearing and Staging

*Toxoneuron nigriceps* wasps were reared on larval stages of its host, *Heliothis virescens*, according to Vinson et al. (1973). *Heliothis virescens* larvae were maintained on a standard artificial diet developed by Vanderzant et al. (1962). Rearing temperature was maintained at 29 ± 1°C for the host, parasitized host larvae and cocoons. *Toxoneuron nigriceps* adults were kept at 25 ± 1°C and fed with water and honey. In both cases, a 16:8 light/dark photoperiod and a relative humidity of 70 ± 5% were adopted. Late 2 or early 3 days old last (fifth) instar larvae of *H. virescens* were individually parasitized by *T. nigriceps* and maintained as described by Pennacchio et al. (1994). *H. virescens* last instar larvae were staged according to Webb and Dahlman (1985) and synchronized as reported by Pennacchio et al. (1992).

### Dissection of Prothoracic Glands

The prothoracic glands (PGs) from parasitized and non-parasitized *H. virescens* 3 days old last instar larvae were dissected in 1X phosphate-buffered saline (PBS) as previously reported (Pennacchio et al., 1998a).

Glands were incubated at 25°C in 100 μl of Grace’s insect medium (Sigma-Aldrich, St. Louis, MO, United States) for 30 min (time of rest) in order to reduce the possibility of their activation by experimental manipulation, as reported for *Manduca sexta* PGs (Bollenbacher et al., 1983; Smith et al., 1986).

### Extraction of Prothoracicotropic Hormone

The brain extract containing PTTH (hereafter referred to as PTTH) was prepared by homogenizing brains dissected from an equal number of *H. virescens* 3 and early 4 days old last instar larvae and stored in ice-cold Grace’s insect medium (Sigma-Aldrich, St. Louis, MO, United States). The homogenate was placed in boiling water for 2 min, cooled to 4°C on ice and centrifuged at 15,000 g at 4°C for 5 min (Pennacchio et al., 1997). Before being used, PTTH extract was filtered with a 0.20 μm Sterile Syringe Filter (Corning Incorporated, Corning, NY, United States) and then diluted in Grace’s insect medium to 0.1 brain equivalent/μl (BE/μl) and either used immediately for the experiments described below or stored at -80°C.

### Analysis of Protein Phosphorylation

The effect of parasitism on the phosphorylation of the TOR targets 4E-BP and S6K was studied in PGs explanted from parasitized and non-parasitized last instar larvae, under six different experimental conditions: basal PGs incubated in Grace’s insect medium (Sigma-Aldrich, St. Louis, MO, United States) from non-parasitized larvae; basal PGs incubated in Grace’s insect medium from parasitized larvae; stimulated PGs with 0.1 BE/μl PTTH from non-parasitized larvae; stimulated PGs with 0.1 BE/μl PTTH from parasitized larvae; PGs from non-parasitized larvae incubated with 1 μM rapamycin (Calbiochem, catalog number 553210, San Diego, CA, United States), a specific inhibitor of TOR; PGs from non-parasitized larvae incubated with 1 μM rapamycin and stimulated with 0.1 BE/μl PTTH.

After dissection and initial incubation (as described above), PGs were incubated for 3 h at 25°C, for all the experimental conditions, except for incubation with rapamycin, in which PGs were pre-incubated with rapamycin alone for 30 min, then transferred to fresh medium containing the same dose of the inhibitor with or without PTTH 0.1 BE/μl.

After the incubation, a pool of 20 PGs for each of these conditions was lysed directly in 2X Laemmli buffer (Laemmli, 1970), to block protease and phosphatase activity. The extracted proteins were separated by 12% polyacrylamide gel electrophoresis and then transferred on a Whatman nitrocellulose membrane (Protran, Dassel, Germany).

Specific antibodies were used to evaluate phosphorylation of the two TOR targets: anti-phospho-4E-BP (Cell Signaling catalog number 2855S, Danvers, MA, United States) and anti-phospho-S6K (Millipore catalog number 04-393, Temecula, CA, United States) (Gu et al., 2012; Scieuzo et al., 2018). Signals obtained with the anti-actin antibody (Abcam, catalog number 75186, Cambridge, United Kingdom) were used as loading controls as reported for other Lepidoptera (Smith et al., 2014). All antibodies were diluted 1:1000 in tris-buffered saline and 0.1% Tween 20 (TBS-T) with 5% bovine serum albumin (BSA), and the incubation was carried out for 16 h. Membranes were sequentially incubated with each of the three antibodies. Goat anti-rabbit conjugated to horseradish peroxidase (Invitrogen, Carlsbad, CA, United States), diluted 1:15,000 in TBS-T, was used as secondary antibody. Detection was carried out using enhanced chemiluminescence (ECL) (LiteAB Blot Kit – Euroclone, Pavia, Italy) and signals were measured by a Chemidoc^TM^ MP System (Bio-Rad, Milan, Italy).

### *In vitro* Biosynthesis of Ecdysone

Following the dissection of PGs, Grace’s insect medium was replaced with a fresh medium containing stimulators or inhibitors, as described in section Analysis of Protein Phosphorylation. Ecdysone released in the medium was determined by a competitive enzyme immunoassay (EIA), using anti-ecdysone as primary antibody and 20-hydroxyecdysone-peroxidase conjugated as tracer, as previously described (Kingan, 1989; Scieuzo et al., 2018). All experiments were performed on a single PG, in three technical replicates for each of the six biological replicates.

### RNA Extraction and Prothoracic Glands *de novo* Transcriptome Assembly

In order to identify genes differentially expressed in *H. virescens* PGs from parasitized and non-parasitized last instar larvae, a transcriptome analysis was conducted. Total RNA from 300 PGs explanted from 3 days old last instar parasitized (48 h post-parasitism) and synchronized non-parasitized larvae, was extracted using TRI-Reagent (Sigma-Aldrich, St. Louis, MO, United States), according to the manufacturer’s protocol. An additional DNase (Turbo DNase, Ambion Inc., Austin, TX, United States) treatment was carried out before the second purification step to remove any remaining DNA. The DNase enzyme was removed, and the RNA was further purified by using the RNeasy MinElute Clean up Kit (Qiagen, Venio, Netherlands), following the manufacturer’s protocol, and eluted in 20 μl of RNA Storage Solution (Ambion Inc., Austin, TX, United States). RNA integrity was verified on an Agilent 2100 Bioanalyzer using the RNA Nano chips (Agilent Technologies, Palo Alto, CA, United States) and RNA quantity was determined by Nanodrop ND-1000 spectrophotometer (Thermo Scientific, Waltham, MA, United States). Poly(A)+ RNA was isolated from 5 μg total RNA for PGs from parasitized and non-parasitized larvae using the Ambion MicroPoly(A) Purist Kit according to the manufacturer’s instructions (Life Technologies, Carlsbad, CA, United States).

Sequencing was carried out by the Max Planck Genome Center^1^ using standard TruSeq procedures on an Illumina HiSeq2500 sequencer, generating approximately 40 mio paired-end (2 × 100 bp) reads for each of the tissue samples. Quality control measures, including the filtering of high-quality reads based on the score given in FASTQ files, removal of reads containing primer/adaptor sequences and trimming of read lengths, were carried out using CLC Genomics Workbench v9.1^2^. The *de novo* transcriptome assembly was carried out with the same software, selecting the presumed optimal consensus transcriptome as previously described (Vogel et al., 2014). All obtained sequences (contigs) were used as query for a BLASTX searches (Altschul et al., 1997) against the non-redundant database of the National Center for Biotechnology Information (NCBI), considering all hits with an *e-*value < 1E-3. The transcriptome was annotated using BLAST, Gene Ontology (GO) and InterPro terms (InterProScan, EBI), enzyme classification (EC) codes, and metabolic pathways (Kyoto Encyclopedia of Genes and Genomes, KEGG) as implemented in BLAST2GO v4. 1^3^. Based on the BLAST hits, the contigs were assigned to either insect or virus (i.e., *Tn*BV) origin. To optimize the annotation of data, we used GO slim, which uses a subset of the complete GO terms that provides a broader overview of the transcriptome ontology content.

### Digital Gene Expression Analysis

Digital gene expression analysis was carried out by using CLC Genomics workbench v9.1 to generate BAM (mapping) files and QSeq Software (DNAStar Inc.) to remap the Illumina reads onto the reference transcriptome and then counting the sequences to estimate expression levels, using previously described parameters for read mapping and normalization (Vogel et al., 2014; Jacobs et al., 2016). In particular, the expression abundance of each conting was calculated based on the reads per kilobase per million mapped reads (RPKM) method (Mortazavi et al., 2008), using the formula:

RPKM (A) = (10,00,000 × C × 1,000)/(N × L), where RPKM (A) is the abundance of gene A, C is the number of reads that uniquely aligned to gene A, N is the total number of reads that uniquely aligned to all genes, and L is the number of bases in gene A. The RPKM method is able to eliminate the influence of different gene lengths and sequencing discrepancy in the calculation of expression abundance.

### Quantitative RT-PCR (qRT-PCR)

To evaluate the relative expression of *tor, 4ebp*, and *s6k* genes in parasitized and non-parasitized *H. virescens* PGs, quantitative RT-PCR (qRT-PCR) experiments were carried out on an ABI PRISM^®^ 7500 Fast Real-Time PCR System Thermal Cycler (Applied Biosystems, Foster City, CA, United States), with cDNA samples prepared from 3 days old last instar parasitized and non-parasitized PGs, following the guidelines reported in Minimum Information Required for Publication of Quantitative Real-Time PCR experiments (MIQE) (Bustin et al., 2009) and minimum information necessary for quantitative real-time PCR experiments (Johnson et al., 2014). Based on their relative expression levels obtained from our RNAseq data showing that five candidate control genes were not affected by parasitization (Supplementary Figure S1), Glyceraldehyde-3-phosphate dehydrogenase (*Gapdh*), elongation factor 1-alpha (*ef1a*) and ribosomal protein L13 (*rp13*) were chosen as reference genes for normalization of qRT-PCR data. Specific primers for each *H. virescens* gene (*tor, s6k, 4ebp*) and reference genes were designed using Primer 3.0^4^ (Supplementary Table S1).

Genes of interest and reference gene sequences were obtained from the PG *de novo* transcriptome assembly (Scieuzo et al., 2018).

PCR amplifications were performed using GoTaq qPCR Master Mix (Promega, Madison, WI, United States). The reactions were carried out in a 20 μl volume containing 20 ng cDNA and 0.3 μmol/L final primer concentration. Cycling conditions for all genes were: 2 min at 95°C, followed by 40 cycles of 15 s at 95°C and 1 min at 58°C. At the end of each run, a melting curve analysis was performed to confirm the specificity of PCR products. All amplification reactions were run in triplicate (technical replicates) and included negative controls (non-template reactions, replacing cDNA with ultrafiltered sterile water). All qRT-PCR analyses were performed for a set of three biological replicates. In order to evaluate gene expression levels, relative quantification was performed using equations described by Liu and Saint (2002), based on PCR amplification efficiencies of reference and target genes. Amplification efficiency of each target gene and endogenous genes was determined according to the equation E = 10^-1/S^ - 1 (Lee et al., 2006), where S is the slope of the standard curve generated from three serial 10-fold dilutions of cDNA. Quantification analysis of amplification was performed using the comparative CT (ΔCT) method. The efficiencies of the amplicons were approximately equal (*gapdh* parasitized = 0.98; *gapdh* non-parasitized = 0.96; *ef* parasitized = 0.82; *ef* non-parasitized = 0.86; *rpl13* parasitized = 0.91; *rpl13* non-parasitized = 0.93; *tor* parasitized = 0.89; *tor* non-parasitized = 0.88; *4ebp* parasitized = 0.97; *4ebp* non-parasitized = 0.94; *s6k* parasitized = 0.88; *s6k* non-parasitized = 0.87).

### Statistical Analysis

qRT-PCR data were expressed as mean ± SEM (standard error of mean) of independent biological replicates and were compared by a one-way analysis of variance (ANOVA) and Bonferroni *post hoc* test using GraphPad Prism 6.00 software for Windows (GraphPad Software, La Jolla, CA, United States^5^). Enzyme immunoassay data were expressed as mean ± SEM (standard error of mean) of independent biological replicates and were compared by a two-way analysis of variance (ANOVA) with Treatment (3 levels: Parasitized, non-parasitized Control and non-parasitized Rapamycin-treated) as the first factor and PTTH-stimulated (2 levels: yes, no) as the second factor. *Post hoc* Means Comparison test was done with both Tukey and SNK tests. In order to correct heteroscedasticity and non-normality, checked with Levene and Shapiro–Wilk tests, data were square root transformed before the analysis. All the statistical analysis was done using Systat 13 (Systat Software, Inc., San Jose, CA, United States^6^).

## Results

### Phosphorylation of 4E-BP and S6K Proteins in Parasitized and Non-parasitized Prothoracic Glands

In order to verify the effect of parasitism on the TOR pathway and a potential impact on *Heliothis virescens* ecdysteroidogenesis, western blot analyses were performed on protein extracts from PGs, previously incubated under different conditions (basal, PTTH, rapamycin, PTTH added with rapamycin) explanted from non-parasitized and parasitized last instar (fifth) larvae. The phosphorylation of the main targets of TOR kinase was detected using antibodies against phospho-4E-BP and phospho-S6K, respectively.

**FIGURE 1:**
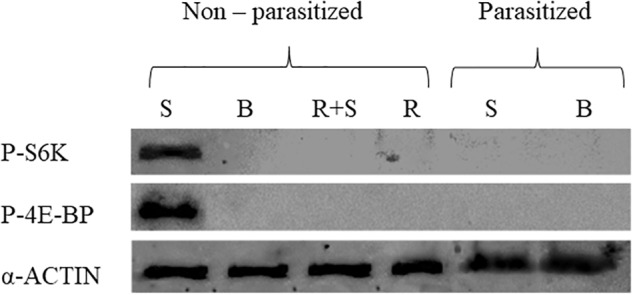
Phosphorylation of TOR target proteins, 4E-BP and S6K, in prothoracic glands, explanted from parasitized and non-parasitized larvae, under different experimental conditions. Basal prothoracic glands (B) or stimulated prothoracic glands with PTTH extract (S), rapamycin (R), explanted from parasitized or non-parasitized larvae. Prothoracic glands (PGs) from 3 days old last instar parasitized and non-parasitized larvae were incubated with 0.1 BE/μl PTTH (stimulated, S). Non-parasitized PGs were also pre-incubated with 1 μM rapamycin for 30 min, then transferred to fresh medium containing the same dose of the inhibitor with (stimulated, R+S) or without (R) 0.1 BE/μl PTTH. Parasitized and non-parasitized (basal, B) glands were incubated in Grace’s insect medium. Incubation was maintained for 3 h in each condition at 25°C. Glands lysed in Laemmli buffer 2X, were analyzed by western blot using antibodies against phospho-4E-BP (20 kDa), phospho-S6K (70 kDa). Each lane represents the equivalent of 20 prothoracic glands. The quantity of loaded proteins was verified by the endogenous control, α-Actin (42 kDa).

The *in vitro* exposure of PGs explanted from 3 days old last instar non-parasitized larvae to PTTH contained in brain extract enhanced the phosphorylation level of both 4E-BP and S6K proteins. No phosphorylation signals were detected in PGs explanted from non-parasitized larvae, treated with rapamycin (with or without PTTH stimulation) and from parasitized larvae (with or without PTTH stimulation) (Figure 1).

### *In vitro* Effect of Parasitism on Ecdysteroidogenesis

**FIGURE 2:**
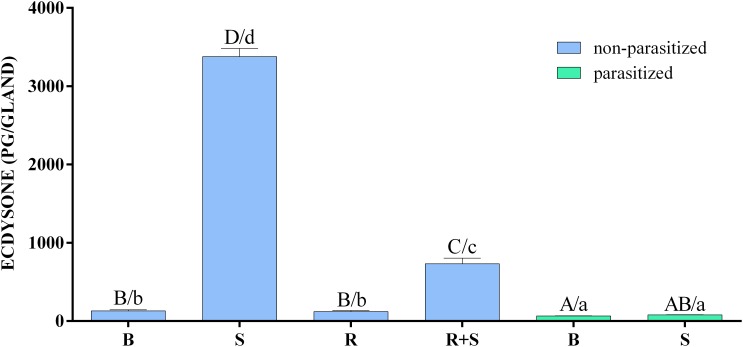
Inhibitory effect of parasitism and rapamycin on the synthesis of ecdysone by prothoracic glands of *H. virescens*. Prothoracic glands (PGs) explanted from parasitized and non-parasitized 3 days old last instar larvae were incubated in Grace’s insect medium with (stimulated, S) or without (basal, B) 0.1 BE/μl PTTH. Non-parasitized PGs were also pre-incubated with 1 μM rapamycin for 30 min, then transferred to fresh medium containing the same dose of the inhibitor with (rapamycin stimulated, R+S) or without (rapamycin basal, B) 0.1 BE/μl PTTH. Parasitized and non-parasitized (basal) glands were incubated in Grace’s insect medium. Incubation was maintained for 3 h in each condition at 25°C, after which the ecdysone produced was determined by enzyme immunoassay (EIA). Data are expressed as mean ± SEM of *n* = 6 experiments. Statistical analysis was performed by a two-way analysis of variance (ANOVA). *Post hoc* Means Comparison test was done with both Tukey and SNK tests. Different letters indicate significant differences (*p* < 0.05). Uppercase letters refer to the Tukey *post hoc* test and lowercase letters to the SNK test.

The *in vitro* biosynthetic activity of PGs explanted from *H. virescens* 3 days old last instar parasitized and non-parasitized larvae, in response to activators or inhibitors, is reported in Figure 2 and Supplementary Table S2.

Ecdysone production was strongly enhanced by PTTH stimulation of non-parasitized PGs in comparison to all other experimental conditions. In the presence of rapamycin, the ecdysone production following PTTH-stimulation, although significantly lower compared to stimulation conditions, was significantly higher than basal or rapamycin-treated non-parasitized PGs, and basal or PTTH-stimulated parasitized PGs. In both PTTH-stimulated and non-stimulated parasitized PGs the titre of ecdysone was lower to that released by basal non-parasitized PGs (Supplementary Table S3).

### Transcriptome Analyses of Parasitized and Non-parasitized (Combined) *H. virescens* Prothoracic Glands

**FIGURE 3:**
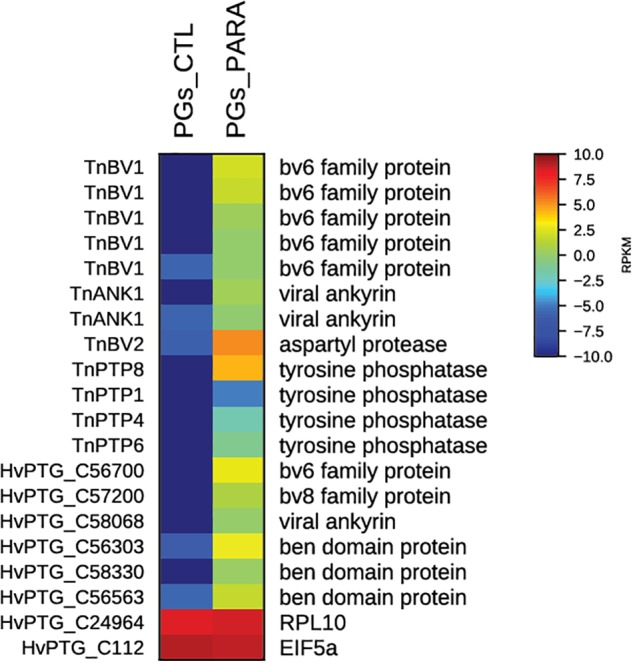
Heat map showing relative expression levels of *Tn*BV genes in PGs from parasitized (PGs_PARA) and non-parasitized (PGs_CTL) *H. virescens* larvae. Viral genes are exclusively expressed in PGs from parasitized larvae. The housekeeping genes *RPl10* and *EIF5a* are used for normalization and are shown to confirm the uniform expression of these control genes across samples. The map is based on log2-transformed RPKM values shown in the gradient heat map (blue represents weakly-expressed genes, and red represents strongly-expressed genes).

For functional annotations, all sequences were subjected to Gene Ontology (GO) analyses in Blast2GO revealing that of the total number of contigs, 61% (40,290) shared significant similarity to proteins with assigned molecular functions in the GO database (Altschul et al., 1997). The analyses of the combined transcriptome allowed the identification of 18 contigs of viral origin selectively transcribed and putatively expressed in parasitized PGs. Among these, 6 contigs could be assigned to general bracovirus genes, while 12 could be assigned specifically to *Tn*BV, as visualized in the heat map with the normalized mapped read (RPKM) counts (Figure 3).

**FIGURE 4:**
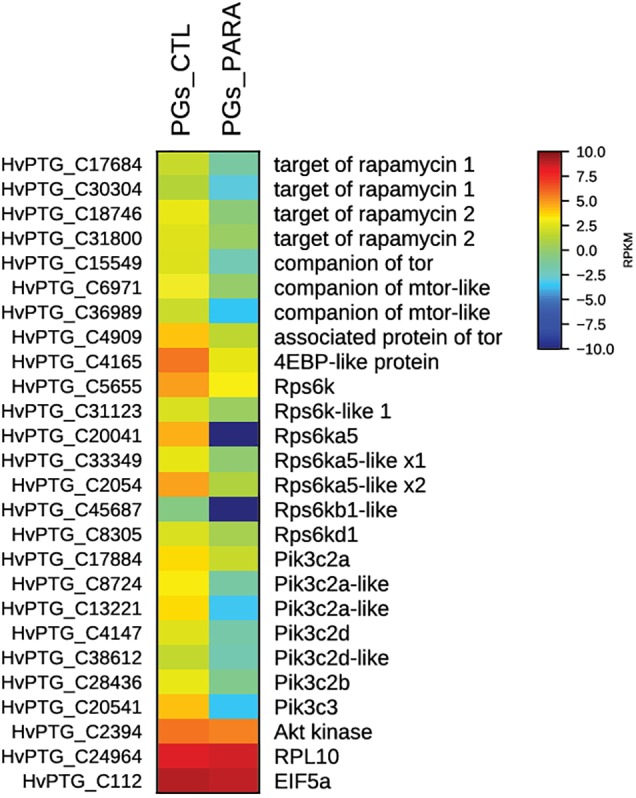
Heat map showing relative expression levels of all TOR pathway genes in PGs from parasitized (PGs_PARA) and non-parasitized (PGs_CTL) larvae. Genes belonging to the TOR pathway are downregulated in parasitized larvae compared to the control (non-parasitized). The housekeeping genes *RPl10* and *EIF5a* are used for normalization and are shown to confirm the uniform expression of these control genes across samples. The map is based on log2^-^ transformed RPKM values shown in the gradient heat map (blue represents weakly-expressed genes, and red represents strongly-expressed genes).

Using the transcriptome and RNAseq mapping data, it was also possible to evaluate the transcript levels of all genes encoding for proteins involved in the cell signaling pathway PI3K/Akt/TOR. As shown in the respective heat map (Figure 4), in PGs extracted from parasitized larvae all of the identified genes are downregulated compared to control (non-parasitized) PGs.

**FIGURE 5:**
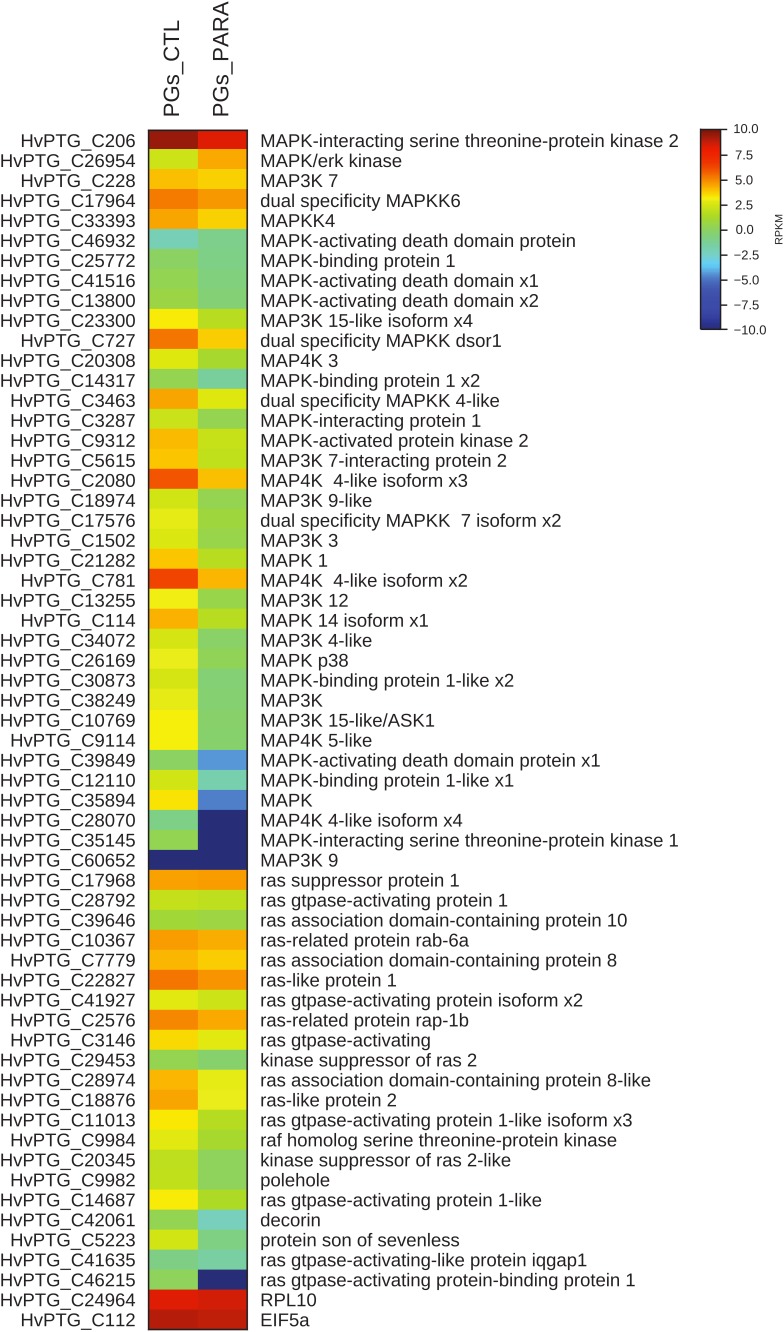
Heat map showing relative expression levels of MAPK pathway genes in PGs from parasitized (PGs_PARA) and non-parasitized (PGs_CTL) larvae. Genes belonging to the MAPK pathway are downregulated in parasitized larvae compared to the control (non-parasitized). The housekeeping genes *RPl10* and *EIF5a* are used for normalization and are shown to confirm the uniform expression of these control genes across samples. The map is based on log2^-^ transformed RPKM values shown in the gradient heat map (blue represents weakly-expressed genes, and red represents strongly-expressed genes).

**FIGURE 6:**
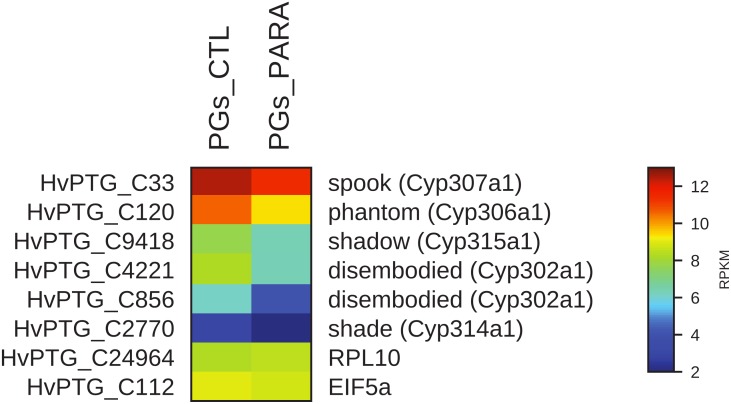
Heat map showing relative expression levels of *Halloween* genes in PGs from parasitized (PGs_PARA) and non-parasitized (PGs_CTL) larvae. *Halloween* genes are downregulated in parasitized larvae compared to the control (non-parasitized). The housekeeping genes *RPl10* and *EIF5a* are used for normalization and are shown to confirm the uniform expression of these control genes across samples. The map is based on log2^-^ transformed RPKM values shown in the gradient heat map (blue represents weakly-expressed genes, and red represents strongly-expressed genes).

Moreover, the transcript levels of genes codifying for proteins involved both in MAPK pathway and in the biosynthesis of ecdysone (*Halloween* genes) were evaluated. In PGs extracted from parasitized larvae, 57 of the 58 identified transcripts encoding for proteins belonging to the MAPK pathway are downregulated compared to control (non-parasitized larvae PGs) (Figure 5). The downregulation of 5 among the 6 identified contigs corresponding to *Halloween* genes is also observed in PGs extracted from parasitized larvae compared to non-parasitized larvae PGs (control) (Figure 6).

### Gene Expression Levels in Parasitized and Non-parasitized Prothoracic Glands

**FIGURE 7:**
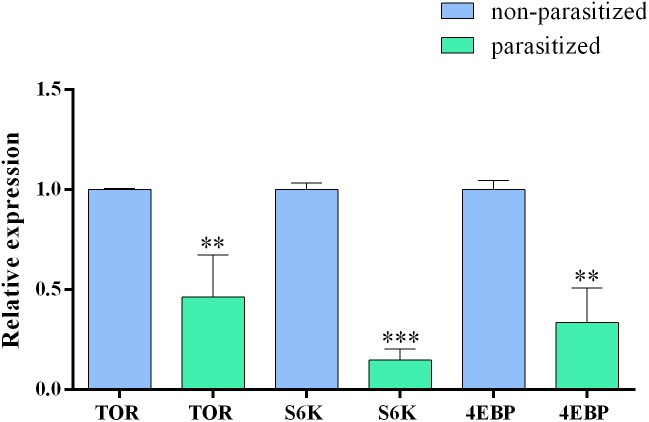
Relative expression level of *tor, s6k*, and *4ebp* genes in basal parasitized and basal non-parasitized PGs. Gene expression levels were quantified by qRT-PCR. Data represent the mean of three independent replicates ± SEM. Statistically significant differences between samples are indicated with asterisk (^∗∗^*p* ≤ 0.01 and ^∗∗∗^*p* ≤ 0.001, one way ANOVA and Bonferroni *post hoc* <0.05). Reference genes: *gapdh, rp13, and ef*. Calibrator sample: non-parasitized PGs.

In order to confirm the transcriptomic analysis, showing a downregulation of TOR pathway genes, we analyzed the relative expression of *tor*, *4ebp*, and *s6k* genes by quantitative Real time PCR (qRT-PCR) in parasitized and non-parasitized PGs. Our results showed that all tested genes displayed lower expression levels in parasitized PGs. The expression levels were statistically significantly different compared to those in non-parasitized samples (Figure 7).

## Discussion

The serine/threonine protein kinase Target of Rapamycin (TOR) is one of the key proteins involved in the control of several cell processes. TOR belongs to the phosphoinositide 3-kinase/protein kinase B (PI3K/Akt) pathway and its activation is modulated by the combination of signaling pathways following intracellular stimuli, such as nutrients and growth factors. The TOR protein and other proteins belonging to its cellular cascade are highly conserved among eukaryotes from yeast to mammals, including insects (Zhang et al., 2000; Rexin et al., 2015; Gonzalez and Hall, 2017). The alteration of the PI3K/Akt pathway, which leads to the overexpression or the inactivation of TOR kinase, seems to be critical in a number of diseases, such as cancer and diabetes (Ali et al., 2017), or neurological diseases such as Parkinson (Xu et al., 2014; Tiwari and Pal, 2017) and Alzheimer (Tang et al., 2015; Tramutola et al., 2015). TOR therefore represents one of the major therapeutic targets in these pathological alterations. The macrolide rapamycin and its analogs are highly specific inhibitors of TOR and they are considered therapeutic molecules with antitumor (Calimeri and Ferreri, 2017; Faes et al., 2017; Liu et al., 2017) and immunosuppressive activity (McMahon et al., 2011; Hamdani et al., 2017; Yee and Tan, 2017), and with promising activity against neurological disease (Wang et al., 2014; Maiese, 2016). These molecules have a unique mechanism of action: they first bind FK506-binding protein (FKBP12), a 12 kDa immunophilin, and then this complex inhibits the serine/threonine kinase TOR (Russell et al., 2011).

The use of rapamycin or its analogs has also side effects: dose-dependent cytopenia, hyperlipidemia, thrombosis and pulmonary, cardiovascular, skin or bone damages (Bhat et al., 2013). The identification of new genes and molecules able to modulate/inhibit the TOR pathway with a similar or different mechanism as rapamycin can be considered an important goal to enrich the strategy and tools employable in cancer therapy or in pathology related to TOR pathway deregulation.

Beside the important roles regarding the regulation of cell growth and proliferation in response to environmental and nutritional conditions, in insects the TOR pathway is involved in ecdysone biosynthesis by PGs or analogous organs, stimulated by the PTTH. In previous work we demonstrated the involvement of the PI3K/Akt/TOR pathway in ecdysteroidogenesis stimulated by PTTH, contained in the brain extract, in *Heliothis virescens* PGs (Scieuzo et al., 2018). Here we show that this cellular signaling pathway is one of the targets of infection by *Tn*BV, the Polydnavirus associated with the endoparasitoid wasp *Toxoneuron nigriceps*.

*T. nigriceps* oviposits into all larval instars of *H. virescens*, which can reach the stage of mature larva (last instar) but become developmentally arrested failing pupation. The PGs functional inactivation is responsible for blocking pupation of the parasitized host last instar larvae (Tanaka and Vinson, 1991; Pennacchio et al., 1997, 1998a,b, 2001). The reduced biosynthetic activity of host PGs was due to their infection by transcriptionally active *Tn*BV, suggesting that viral gene expression in PGs might play a role in the disruption of the PTTH signal transduction pathway (Pennacchio et al., 1998a).

Our recent studies showing a PI3K/Akt/TOR signaling involvement in PTTH-stimulated ecdysteroidogenesis by *H. virescens* PGs, confirmed that PTTH rapidly enhanced the phosphorylation of translational repressor 4E-binding protein (4E-BP) and p70 ribosomal protein S6 kinase (S6K), two well-known downstream targets of TOR. Moreover, we also demonstrated that rapamycin blocked phosphorylation of 4E-BP and S6K in PTTH-stimulated PGs and strongly inhibited PTTH-stimulated ecdysteroidogenesis (Scieuzo et al., 2018).

In the present study the possible role of *Tn*BV on the PI3K/Akt/TOR pathway in PTTH-stimulated ecdysone biosynthesis in *H. virescens* PGs was investigated. Interestingly, our results confirm that a parasitism event completely inhibits PTTH-mediated stimulation of ecdysone biosynthesis in PGs of parasitized larvae, as suggested in previous work (Pennacchio et al., 1997, 1998a; Falabella et al., 2006), and demonstrate that one of the *Tn*BV effects is linked to PI3K/Akt/TOR pathway alteration. The impact of *Tn*BV on ecdysteroidogenesis is more dramatic than the effect of rapamycin: parasitism totally inhibited ecdysone production of PTTH-stimulated PGs, whereas the effect of rapamycin was partial. This difference can be explained on the basis of our previous study (Scieuzo et al., 2018) and other studies on lepidopteran species (Lin and Gu, 2007; Gu et al., 2011) which demonstrated that both MAPK and PI3K/Akt/TOR pathways are independently involved in PTTH-stimulated ecdysteroidogenesis, but rapamycin only affects the TOR pathway. Evidently the expression of *Tn*BV genes in *H. virescens* PGs affects PTTH-stimulated ecdysteroidogenesis pathways at different levels and also with different mechanisms. Moreover, the PTTH-stimulated phosphorylation of 4E-BP and S6K, detected only in non-parasitized PGs, indicates that the PI3K/Akt/TOR pathway is directly stimulated by the neuropeptide hormone, as previously demonstrated (Scieuzo et al., 2018); no phosphorylation signal was detected in parasitized PGs, both basal and stimulated, apparently similar to the effect of PG incubation with rapamycin (Gu et al., 2011, 2012; Scieuzo et al., 2018). *In vitro* ecdysone biosynthesis evaluation showed that non-parasitized PGs, treated with rapamycin (R) and stimulated with PTTH extract (R+S), produced a significantly lower amount of ecdysone in comparison to non-parasitized PGs stimulated with PTTH extract (S), but a significantly higher amount of ecdysone in comparison to both untreated parasitized PGs (B) and those stimulated with PTTH extract (S), confirming that a parasitism event completely blocks ecdysteroidogenesis. This confirms, above all, that PI3K/Akt/TOR is not the only pathway involved in *H. virescens* ecdysteroidogenesis, suggesting that parasitization affects all the signaling pathways involved in ecdysteroidogenesis (Scieuzo et al., 2018).

Taken together, our results indicate that the infection of host PGs by *Tn*BV alters ecdysone production, at least in part, by modulating the TOR pathway through the expression of one or more viral genes. In support of this hypothesis, we identified all viral genes expressed in PGs, comparing the expression levels of transcripts in parasitized and non-parasitized PGs. Among these were previously identified and, in some cases, functionally characterized *Tn*BV genes (Varricchio et al., 1999; Falabella et al., 2003; Provost et al., 2004) such as *TnBV1*, *TnBV2*, *TnBVank1*, *ptp1*, *ptp4*, *ptp6*, and *ptp8*. *Tn*BVank1 displays significant sequence similarity with members of the IkB family (Silverman and Maniatis, 2001; Thoetkiattikul et al., 2005; Falabella et al., 2007; Bitra et al., 2012; Salvia et al., 2017). These proteins are generally involved in the control of NF-kB signaling pathways both in insects and vertebrates (Silverman and Maniatis, 2001).

Using *Drosophila melanogaster* as a model to functionally characterize *Tn*BV genes, it was shown that *Tn*BVank1 expression in host germ cells altered the microtubule network in oocytes (Duchi et al., 2010; Valzania et al., 2014). Subsequently, Valzania et al. (2014) confirmed that the expression of *Tn*BVank1 in PG cells strongly reduced ecdysone biosynthesis and, as a consequence, inhibited the transition of *D. melanogaster* larval to pupal stage, mimicking the developmental arrest observed in *H. virescens* larvae parasitized by *T. nigriceps*.

These results support the hypothesis that *Tn*BVank1, expressed in *H. virescens* PGs, could actively participate in inducing ecdysone titer reduction. *Tn*BVank1 influences different physiological pathways involved in both the disruption of cytoskeletal structure of PG cells and in affecting the sterol delivery from endosomal compartments trough the interaction with Alix, as reported for *D. melanogaster* (Valzania et al., 2014). This multifunctional activity of *Tn*BVank1 is not surprising. The expression of this gene in *H. virescens* immune cells was demonstrated to induce apoptosis through the interaction with Alix, besides its irreversible inhibition of NF-kB translocation in cell nuclei, thus blocking the expression of key genes and inducing apoptotic phenomena (Falabella et al., 2007; Salvia et al., 2017). The reduced gland size observed in *D. melanogaster* larvae (Valzania et al., 2014) and the low basal production of ecdysteroids in PGs of *H. virescens* parasitized larvae were also reported (Pennacchio et al., 1997, 1998b). Here we demonstrate that in naturally parasitized larvae these symptoms were associated with a disruption of PTTH signaling by active *Tn*BV infection of PGs (Pennacchio et al., 1998a). It is highly plausible that at least part of these effects could be attributable to the expression of *Tn*BVank1 in the parasitized host.

Although the effects of other viral genes should be analyzed *in vivo* or *in vitro*, we can speculate on possible roles of different *Tn*BV genes in the suppression of ecdysteroidogenesis.

Among the 13 putative PTPs identified in the *Tn*BV genome, 8 PTP genes have a full protein tyrosine phosphatase domain (Provost et al., 2004; Falabella et al., 2006).

Our results demonstrate that PTP1, 4, 6, and 8 are specifically expressed in parasitized PGs. These PTPs together with PTP7 (Provost et al., 2004; Falabella et al., 2006), could dephosphorylate PG proteins phosphorylated by tyrosine kinases following PTTH stimulation. The expression of *Tn*BV PTP7 (24 h after parasitism) (Falabella et al., 2006) and of *Tn*BV PTP1, PTP4, PTP6, and PTP8 (reported here) specifically in PGs, confirms that PTP expression is host tissue-specific.

Members of the *Tn*BV PTP gene family, expressed at different times during parasitism, could be good candidates for a functional involvement in host PG inactivation through dephosphorylation of regulatory proteins. These proteins are possibly involved in the extremely intricate PTTH-stimulated ecdysone secretion pathway. However, at least in case of *H. virescens* and other Lepidoptera, this pathway is not yet fully identified. Since *Tn*BV PTPs belong to the classical PTPs (Provost et al., 2004), they dephosphorylate only tyrosine residues, and it is reasonable to hypothesize that *Tn*BV PTPs do not directly affect the TOR pathway (Brunn et al., 1997; Aoki et al., 2001). The target/s of viral PTPs still remain/s unknown.

The effect of parasitism on PGs could act on different levels to ensure the total inhibition of PG biosynthetic activity. Indeed, we demonstrate an alternative way to control the TOR pathway at the transcriptional level through expression of one or more *Tn*BV genes in PGs of parasitized larvae. The down-regulation of TOR genes, especially *tor, s6k*, and *4ebp*, was confirmed by qRT-PCR experiments. Although it remains unclear which of the *Tn*BV genes are involved, *TnBVank1* could play a role, also because of its ability to block NF-kB-mediated gene expression regulation (Falabella et al., 2007; Dan et al., 2008; Oeckinghaus et al., 2011).

The expression of *Tn*BV genes in PGs seems to alter TOR metabolic pathway, influencing essential steps for the synthesis of ecdysone. However in *H. virescens* at least two independent pathways contribute to ecdysteroidogenesis: MAPK and PI3K/Akt/TOR cellular signaling (Scieuzo et al., 2018). RNA-seq data showed a down-regulation of genes involved in both PI3K/Akt/TOR, MAPK pathways, and in the biosynthesis of ecdysone (*Halloween* genes). These findings suggest that the massively reduced amounts of ecdysone following *T. nigriceps* parasitism could be ascribed to the expression of *Tn*BV genes in PGs. Further studies are needed to obtain more information regarding the possible mechanism of action of *Tn*BV proteins on these pathways. Further characterization of other viral proteins would allow a better understanding of the mechanisms involved in the inhibition of ecdysone synthesis, and could provide a range of candidates potentially capable of inhibiting more steps of the PI3K/Akt/TOR pathway. The possible use of viral proteins in synergy with rapamycin or its analogs can be a turning point in medical treatment with the PI3K/Akt/TOR pathway as possible therapeutic target.

## Data Availability

The short read data have been deposited in the EBI short read archive (SRA) with the following sample accession numbers: ERS2859514-ERS2859515 (PGs of non-parasitized *H. virescens* larvae) and ERS2859516 (PGs of *T. nigriceps* parasitized *H. virescens* larvae). The complete study can also be accessed directly using the following URL: http://www.ebi.ac.uk/ena/data/view/PRJEB29401.

## Ethics Statement

Insects used in this work were treated as well as possible given the constraints of the experimental design.

## Author Contributions

PF designed the experiments, wrote and critically revised the paper. HV, RS, MN, CS, AS, AR, and SB contributed to the data interpretation and critically revised the paper. RS and CS performed the western blot experiments. AS, MN, and RS performed the samples collection and RT-qPCR. MN and CS performed the enzyme immunoassay. HV performed the *de novo* transcriptome assembly and analysis. All authors read and approved the manuscript.

## Conflict of Interest Statement

The authors declare that the research was conducted in the absence of any commercial or financial relationships that could be construed as a potential conflict of interest.

## Footnotes

1 http://mpgc.mpipz.mpg.de/home/

2 http://www.clcbio.com

3 http://www.blast2go.de

4 http://bioinfo.ut.ee/primer3-0.4.0

5 www.graphpad.com

6 www.systatsoftware.com

## Supplementary Material

FIGURE S1 Heat map showing relative expression levels of five candidate reference genes in PGs from parasitized (PGs_PARA) and non-parasitized (PGs_CTL) larvae. Eukaryotic translation initiation factor 5A-1 (*eif5a*), ribosomal protein L10 (*rpl10*), Glyceraldehyde-3-phosphate dehydrogenase (*Gapdh)*, elongation factor 1-alpha (*ef1a)* and ribosomal protein L13 (*rp13)* were pre-selected as candidate reference genes for normalization of qRT-PCR data since they were not affected by parasitization. *Gapdh, ef1a* and *rp13* were subsequently chosen as reference genes.

TABLE S1 Primers used for qRT-PCR. F: forward, R: reverse.

TABLE S2 Ecdysone released by prothoracic glands in different experimental conditions. Data are expressed as mean of ecdysone concentrations (pg/gland) ± SEM of n = 6 experiments. Different letters indicate significant differences (*p* < 0.05). Uppercase letters refer to the Tukey *post hoc* test and lowercase letters to the SNK test.

TABLE S3 Raw data of enzyme immunoassay (EIA) **(a)** and Two-Way ANOVA statistical output **(b)**.

## Abbreviations

4E-BP: translational repressor 4E-binding protein
Akt: protein kinase B
ANOVA: analysis of variance
BE: brain equivalent
BLAST: basic local alignment search tool
BSA: bovine serum albumin
ECL: enhanced chemo luminescence
EIA: enzyme immunoassay
GO: Gene Ontology
NCBI: National Center for Biotechnology Information
PBS: phosphate-buffered saline
PDV: polydnavirus
PG: prothoracic gland
PI3K: phosphoinositide 3-kinase
PTP: protein tyrosine phosphatase
PTTH: prothoracicotropic hormone
S6K: p70 ribosomal protein S6 kinase
SEM: standard error of mean
TBS-T: tris-buffered saline-Tween 20
*Tn*BV: *Toxoneuron nigriceps* bracovirus
TOR: target of rapamycin
