# Supplementary figures and images for: Novel Factors of Viral Origin Inhibit TOR Pathway Gene Expression
Source: Front Physiol. 2018 Nov 26;9:1678. doi: 10.3389/fphys.2018.01678 (PMC6275226; doi:10.3389/fphys.2018.01678)

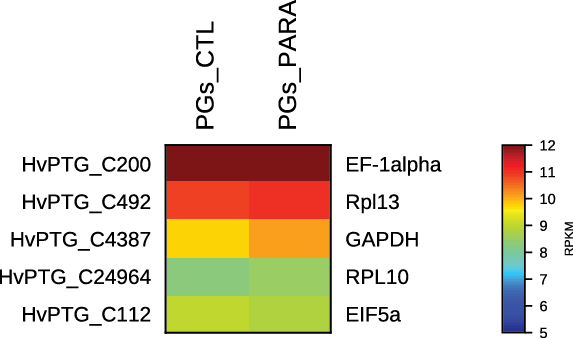

Supplement: FIGURE S1 — Heat map showing relative expression levels of five candidate reference genes in PGs from parasitized (PGs_PARA) and non-parasitized (PGs_CTL) larvae. Eukaryotic translation initiation factor 5A-1 (eif5a), ribosomal protein L10 (rpl10), Glyceraldehyde-3-phosphate dehydrogenase (Gapdh), elongation factor 1-alpha (ef1a) and ribosomal protein L13 (rp13) were pre-selected as candidate reference genes for normalization of qRT-PCR data since they were not affected by parasitization. Gapdh, ef1a and rp13 were subsequently chosen as reference genes. [file Image_1.TIFF]
