# Supplementary material for: Novel Factors of Viral Origin Inhibit TOR Pathway Gene Expression
Source: Front Physiol. 2018 Nov 26;9:1678. doi: 10.3389/fphys.2018.01678 (PMC6275226; doi:10.3389/fphys.2018.01678)
Supplement: TABLE S1 — Primers used for qRT-PCR. F: forward, R: reverse. [file Table_1.DOCX]

| GENE NAME | PRIMER SEQUENCE 5’-3’ |
| --- | --- |
| *tor* | F: GCGGACAGAGATTGGTAGA  R: TGGGTAACGGCATGTTCT |
| *4e-bp* | F: ATGCCCGATGTCTACTCCA  R: TAGGGTTCTTCAGTAATGC |
| *s6k* | F: ATTGGTGGAGTCTTGGAGC  R: GTATCAAATCACGCGCGTG |
| *gapdh* | F: GTCGTTGATCTGACCGTTCG  R: CGTTGTCGTACCAGCTGATG |
| *ef* | F: TTGAAGCCTGGTACCATCGT  R: TTGAAGCCTGGTACCATCGT |
| *rp13* | F: TCGTGGTAAGGTGAAGGCAT  R: AGTCACAGCCTCAACGATCT |
