# Supplementary material for: Novel Factors of Viral Origin Inhibit TOR Pathway Gene Expression
Source: Front Physiol. 2018 Nov 26;9:1678. doi: 10.3389/fphys.2018.01678 (PMC6275226; doi:10.3389/fphys.2018.01678)
Supplement: TABLE S2 — Ecdysone released by prothoracic glands in different experimental conditions. Data are expressed as mean of ecdysone concentrations (pg/gland) ± SEM of n = 6 experiments. Different letters indicate significant differences (p < 0.05). Uppercase letters refer to the Tukey post hoc test and lowercase letters to the SNK test. [file Table_2.docx]

|  | **Ecdysone concentration (pg/gland)** | |
| --- | --- | --- |
|  | Non stimulated | Stimulated |
| Parasitized | 66.28 ± 1.44 ^A/a^ | 81.18 ± 1.99 ^AB/a^ |
| Non parasitized | 131.03 ± 13.69 ^B/b^ | 3378.01 ± 104.83 ^D/d^ |
| Non parasitized, rapamycin treated | 122.59 ± 13.79 ^B/b^ | 733.34 ± 29.03 ^C/c^ |
