# Supplementary material for: Novel Factors of Viral Origin Inhibit TOR Pathway Gene Expression
Source: Front Physiol. 2018 Nov 26;9:1678. doi: 10.3389/fphys.2018.01678 (PMC6275226; doi:10.3389/fphys.2018.01678)
Supplement: TABLE S3 — Raw data of enzyme immunoassay (EIA) (a) and Two-Way ANOVA statistical output (b). [file Table_3.DOCX]

a)

| Non parasitized | | | | Parasitized | |
| --- | --- | --- | --- | --- | --- |
| B | S | R | R+S | B | S |
| 80,00 | 3769,64 | 112,12 | 698,1500 | 67,5742 | 80,2160 |
| 98,64 | 3044,63 | 118,74 | 712,9900 | 68,3882 | 85,9829 |
| 141,28 | 3366,11 | 136,05 | 758,6800 | 68,1210 | 86,7922 |
| 151,63 | 3325,07 | 179,42 | 797,0500 | 69,5314 | 80,4499 |
| 151,89 | 3557,88 | 77,26 | 812,4205 | 63,7883 | 80,3361 |
| 162,72 | 3205,24 | 111,97 | 620,7300 | 60,2533 | 73,3265 |

b)

| Source | Type III SS | df | Mean Squares | F-Ratio | p-Value |
| --- | --- | --- | --- | --- | --- |
| Treatment | 4158.32 | 2 | 2079.16 | 1094.23 | < 0.001 |
| PTTH-Stimulated | 4052.92 | 1 | 4052.92 | 2132.00 | < 0.001 |
| Treatment x Stimulated | 3274.10 | 2 | 1637.05 | 861.56 | < 0.001 |
| Error | 57.00 | 30 | 1.90 |  |  |
